# Translational control of gene expression by eIF2 modulates proteostasis and extends lifespan

**DOI:** 10.18632/aging.203018

**Published:** 2021-04-26

**Authors:** Tamara Jiménez-Saucedo, Juan José Berlanga, Miguel Rodríguez-Gabriel

**Affiliations:** 1 Centro de Biología Molecular Severo Ochoa (CSIC-UAM), Universidad Autónoma de Madrid, Madrid, Spain

**Keywords:** longevity, translational control, autophagy, eIF2 factor, gene expression

## Abstract

Although the stress response in eukaryotes depends on early events triggered in cells by environmental insults, long-term processes such as aging are also affected. The loss of cellular proteostasis greatly impacts aging, which is regulated by the balancing of protein synthesis and degradation systems. As translation is the input event in proteostasis, we decided to study the role of translational activity on cell lifespan. Our hypothesis was that a reduction on translational activity or specific changes in translation may increase cellular longevity.

Using mutant strains of *Schizosaccharomyces pombe* and various stress conditions, we showed that translational reduction caused by phosphorylation of eukaryotic translation initiation factor 2 (eIF2) during the exponential growth phase enhances chronological lifespan (CLS). Furthermore, through next-generation sequence analysis, we found eIF2α phosphorylation-dependent translational activation of some specific genes, especially those involved in autophagy. This fact, together with the observed regulation of autophagy, points to a conserved mechanism involving general and specific control of translation and autophagy as mediators of the role of eIF2α phosphorylation in aging.

## INTRODUCTION

Cellular aging is defined as the progressive reduction in capacity to perform cellular functions, eventually leading to cell death. Replicative and chronological aging in various eukaryotic organisms display strong conservation in their biochemical properties [1]. In yeast, aging can be studied through the measurement of either replicative lifespan [2], defined as the number of divisions a cell can carry out, or chronological lifespan (measured in time units), the period of time a cell population can survive in a post-mitotic state. After dividing exponentially, nutrients in the extracellular medium decrease and cells prepare to enter a quiescent state, known as stationary phase, in which cells are metabolically active but do not divide [3]. During the stationary growth phase, which is comparable to the G0 stage of the cell cycle in higher eukaryotes, fission yeast cells are metabolically active and they can enter and exit stationary phase depending on nutrient availability. Thus, after several days of growth in liquid media, cells are able to divide when plated onto rich medium plates [4]; reduction in this capacity is defined as chronological aging. Thus, the onset of stationary phase is considered the basis of chronological aging, triggering signaling pathways that allow cells to prolong their lifespan. This state is characterized by deprivation of nutrients such as glucose or nitrogen source, so the main activated pathways are related to nutrient signaling as well as to the stress response.

Cell homeostasis is achieved by balancing biosynthesis and turnover mechanisms. Thus, intracellular protein balance depends not only on protein synthesis control, but also on degradation systems like autophagy. Autophagy promotes the bulk removal and degradation of unfolded proteins or aggregates as well as damaged organelles, avoiding an overloaded protein homeostasis system and thus, promoting cell longevity [5].

During normal aging, translational activity is actively reduced, and mutations that interfere with this reduction decrease lifespan. Conversely, mutations or treatments that limit translation can inhibit the normal aging process. Previous studies in *Saccharomyces cerevisiae* have shown that dietary restriction, reduced 60S subunit abundance and Gcn4 expression extend cellular lifespan by similar mechanisms [6, 7]. This reduced translational activity would improve the capacity of the protein homeostasis (proteostasis) network to eliminate aberrant proteins, leading to improved cellular health and longevity.

One of the main mechanisms by which cells control translation is the reversible phosphorylation of the eukaryotic translation initiation factor 2 (eIF2). Under different stress conditions, eIF2 is phosphorylated and acquires a higher affinity for eIF2B, sequestering it in an inactive complex which leads to a huge decline in ternary complex levels, significantly reducing global protein synthesis. Paradoxically, in this situation certain mRNAs (encoding stress response proteins) which contain special features in their 5’ untranslated region (5’UTR) are actively translated [8]. As both biochemical functions, general and specific, take place concomitantly, it might be difficult to assign independent functions to each of them in the aging process. Specific phosphorylation on the conserved serine 51 of the α-subunit of eIF2 (eIF2α) is carried out by a family of serine/threonine kinases with four members (GCN2, HRI, PKR, PERK), known as eIF2α kinases, in response to a wide variety of environmental stresses. In the fission yeast *Schizosaccharomyces pombe* there are three eIF2α kinases (Gcn2, Hri1 and Hri2) able to phosphorylate eIF2α (serine 52 in fission yeast) in response to oxidative stress, heat shock, ultraviolet radiation, nutrient deprivation, etc. [9, 10].

Our main objective was to demonstrate that eIF2-mediated protein homeostasis, also known as proteostasis, including synthesis and degradation processes, has a critical impact in lifespan. Using the fission yeast *Schizosaccharomyces pombe* as a model organism and cells unable to phosphorylate eIF2 subjected to various stress conditions and pharmacological treatments, we have measured either, protein synthesis rate and chronological aging. Furthermore, through next generation sequencing (NGS) in aging-promoting conditions, we have also analysed the changes in specific translation caused by eIF2 phosphorylation. Finally, and supported by NGS results, we assayed the effect of eIF2 phosphorylation in the onset of autophagy, as a main process involved in proteostasis. All together our results uncover the essential role of the phosphorylation of eIF2 on chronological aging through the control of global and specific protein synthesis and autophagy.

## RESULTS

### eIF2α phosphorylation promotes extension of cell lifespan

**Figure 1:**
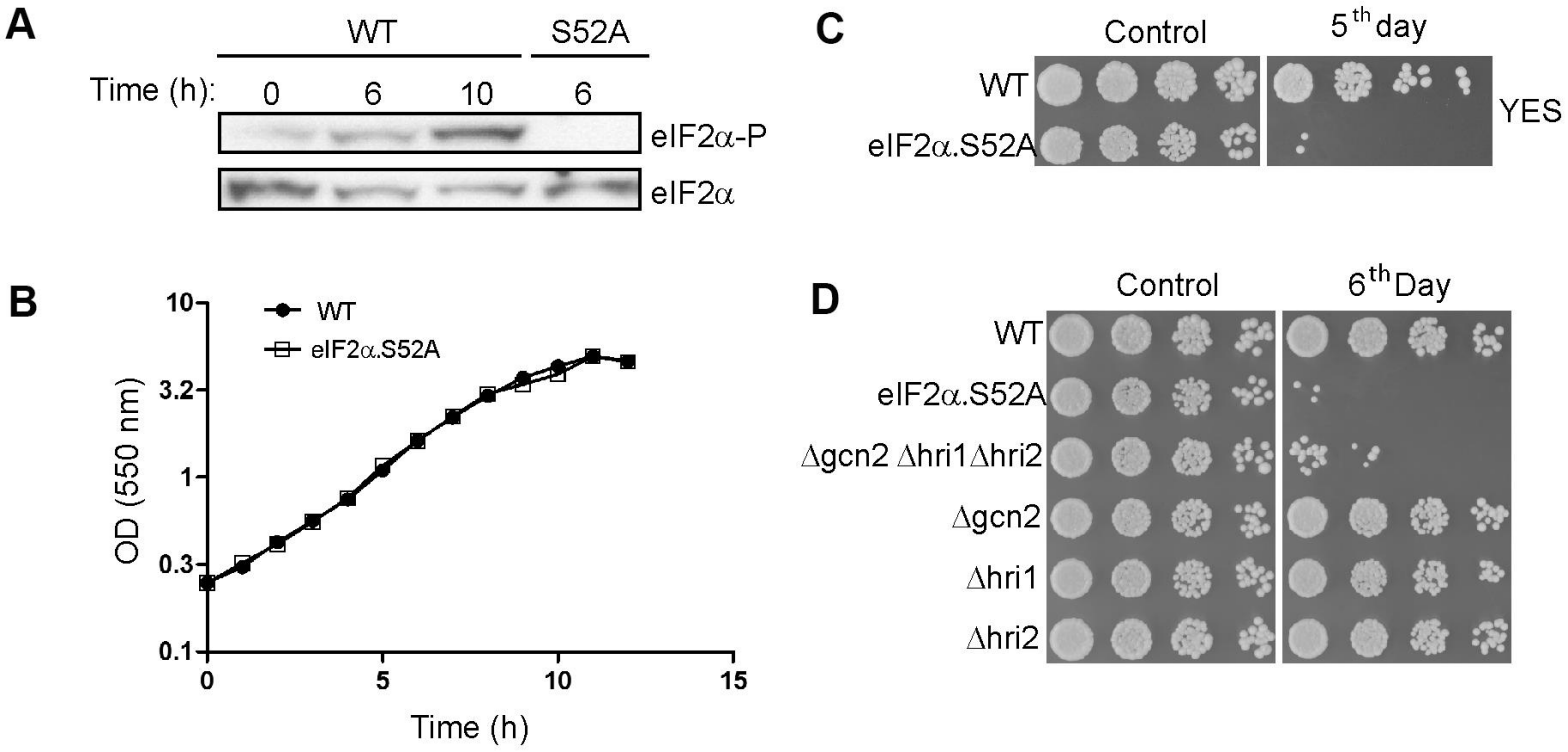
**eIF2α phosphorylation is a key factor in the chronological lifespan of *S. pombe*.** (**A**) eIF2α phosphorylation during the transition from exponential to stationary growth phase. Wild type (WT) and eIF2α.S52A (S52A) cells growing in exponential phase (OD_550_ = 0.5-1) were collected at different time points, as indicated. eIF2α phosphorylation (eIF2α-P) and total amount of eIF2α were detected by western blot. (**B**) Growth curve of WT and eIF2α.S52A cells during exponential growth phase and the onset of stationary phase. Measurement of culture density (OD_550_) is represented in logarithmic scale against time. (**C**, **D**) Serial dilutions (1/5) of different *S. pombe* strains growing in YES medium at the exponential phase (Control) or five (**C**) or six (**D**) days after reaching stationary phase, as indicated, were plated onto YES-Agar plates and were incubated at 30° C for 2-3 days. Data information: (**A**–**D**) Representative results from at least three independent experiments.

In order to understand its possible role in cellular lifespan, we monitored eIF2α phosphorylation state in cells growing exponentially and during their transition to stationary phase. eIF2α phosphorylation increased over time, reaching a high level after 10 h of growth (Figure 1A), as cells proceeded from the exponential to stationary phase (Figure 1B). As a negative control we used a mutant eIF2α strain (eIF2α.S52A) unable to phosphorylate this factor at position 52 due to the presence of an alanine instead of the conserved serine. This result demonstrates that entering stationary phase during chronological aging is a process that occurs along with eIF2α phosphorylation, although phosphorylation does not affect growth rate or the timing of entry into stationary phase, as eIF2α.S52A mutant cells showed similar growth curve to wild type (WT) (Figure 1B).

To determine the role of eIF2α phosphorylation in chronological aging we checked the ability of WT and eIF2α.S52A cells to divide while growing at exponential phase (OD_550_ = 0.5-1) or after several days in stationary phase (Figure 1C). The capacity of WT cells to form colonies was retained after five days of growth, unlike eIF2α.S52A cells, suggesting that eIF2α phosphorylation has a positive effect on longevity in *S. pombe*.

To study the role of the kinases responsible for eIF2α phosphorylation in the aging process, we carried out longevity assays using mutant strains lacking the three eIF2α kinases, with WT and eIF2α.S52A cells used as controls (Figure 1D). We did not find any relevant reduction in longevity among the single kinase mutants. However, cells lacking all three kinases showed an accelerated aging phenotype compared to WT cells, and were very similar to eIF2α.S52A cells. Although none of the eIF2α kinases alone presented a specific role in fission yeast aging, the results support a general effect of eIF2α phosphorylation on aging.

### eIF2α phosphorylation down-regulates translation before quiescence

**Figure 2:**
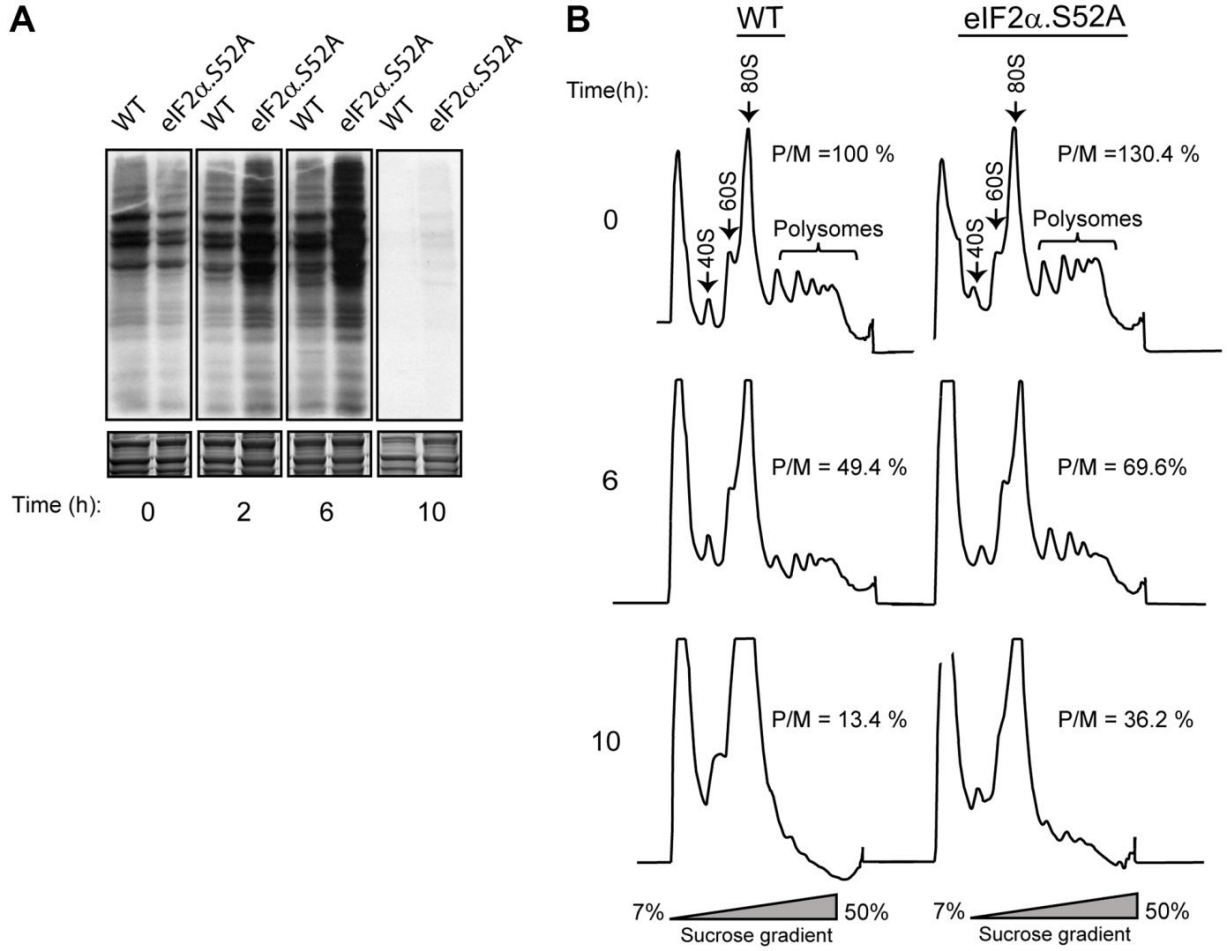
**Phosphorylation of eIF2α plays a key role in protein translation.** (**A**) Metabolic labelling by ^35^S-methionine/cysteine incorporation in WT and eIF2α.S52A cells grown in YES medium. Cells were cultivated to exponential growth phase (time 0) or later time points, as indicated, and labelled for 10 min prior to harvesting. Labelled proteins present in cell extracts were visualized by autoradiography after SDS-PAGE separation (top panel). Coomassie staining shows the total protein levels loaded in each lane (bottom panel). (**B**) Polysome profile analysis of WT and eIF2α.S52A cells. Cell extracts were separated in 7-50 % sucrose gradients and fractioned as described in the Materials and Methods. Polysome/monosome ratios (P/M) were quantified and are shown for each profile. Values are expressed as %, considering the 100% value for WT cells at time = 0, and represent the mean value of three independent experiments. Data information: (**A**, **B**) Representative results from at least three independent experiments.

eIF2α phosphorylation under stress conditions decreases global translation rates while promoting translation of specific mRNAs [11]. We measured global protein synthesis state during exponential growth in WT cells and in cells unable to phosphorylate eIF2α to further investigate the effects of such phosphorylation in critical cellular processes. Translation rate was estimated by incorporation of radioactive amino acids (^35^S-methionine/cysteine) into newly synthesized proteins. Cells maintain an active translation until they start entering stationary phase (6-10 hours of growth) (Figure 2A). Moreover, total incorporation of radioactivity into proteins was higher in eIF2α.S52A cells than in WT cells, indicating a greater translation activity in these mutant cells. However, there was also a strong decline in ^35^S incorporation after 10 h of growth in both cell types, suggesting the existence of additional translation inhibition processes independent of eIF2α phosphorylation at the onset of stationary phase.

We also monitored fission yeast translation rate by polysome profile observation (Figure 2B). In both WT and eIF2α.S52A cells, the polysome/monosome-80S (P/M) ratio decreased over time until reaching the stationary phase and the decline was more evident in WT cells. Both experimental approaches confirmed that eIF2α phosphorylation implements a reduction in translation as the cells advance into stationary phase.

### Nutrient levels and incubation temperature affect translation rates and modulate chronological lifespan

The above results demonstrated a strong correlation between eIF2α phosphorylation, reduced translational activity and extension of lifespan. Thus, we hypothesized that conditions leading to reduced translation should increase cell longevity. To test this, we performed longevity experiments in conditions where cells present a slower or faster metabolism in order to see their causal effects on cell survival.

**Figure 3:**
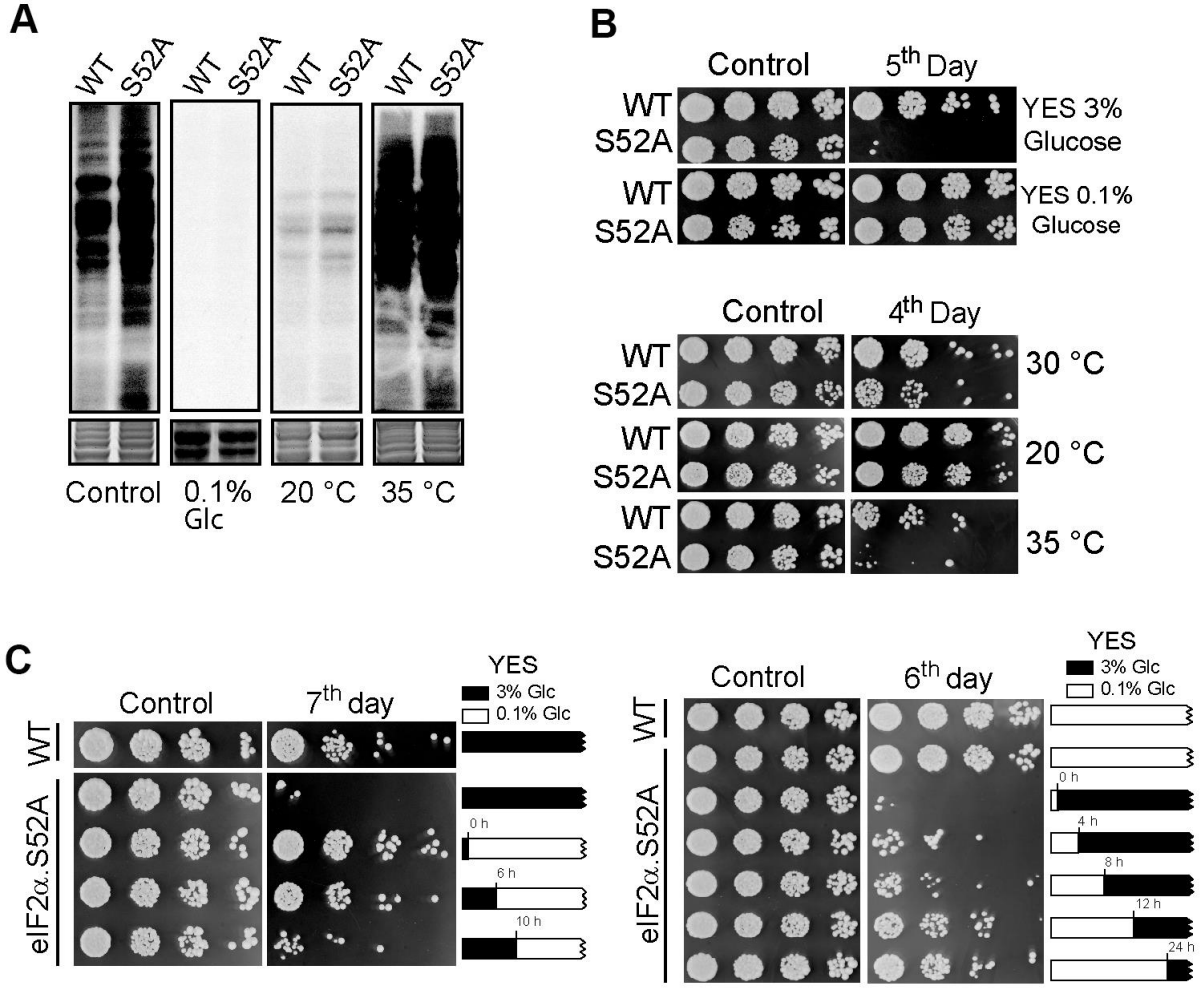
**Effect of nutrient deprivation and temperature on translation and lifespan.** (**A**) Translation rate of WT and eIF2α.S52A (S52A) cells measured by ^35^S-methionine/cysteine metabolic labelling. Coomassie staining shows the total protein levels loaded in each lane (bottom panel). Cells were grown in standard conditions (3% glucose YES medium at 30° C, Control) or low glucose (0.1% Glc) YES medium and at 20° C or 35° C prior to labelling. Cells were metabolically labelled, as previously described. (**B**) Longevity assay of WT and eIF2α.S52A (S52A) cells cultivated under conditions described in A). Serial dilutions (1/5) of cells were plated onto YES-Agar plates at the exponential phase (Control) or several days after reaching stationary phase of growth, as indicated, and incubated at 30° C for 2-3 days. (**C**) Longevity assay of cells shifted from 3% to 0.1% glucose YES medium during exponential growth phase. Left panel: eIF2α.S52A cells growing in 3% glucose (black boxes) were shifted to 0.1% glucose YES medium (white boxes) at indicated times. Right panel: eIF2α.S52A cells growing in 0.1% glucose YES medium were shifted to 3% glucose by addition of glucose at the indicated times. WT and eIF2α.S52A cells grown in 3% or 0.1% glucose YES medium during the whole experiment were used as control. Serial dilutions of cells were plated as described in panel (**B**). Data information: (**A**–**C**) Representative results from three independent experiments.

Caloric restriction is a well-established environmental cue that increases longevity [12]. In fission yeast, it can be achieved by reducing glucose in regular rich growth medium from 3% to 0.1%. At the same time, incubation at 20° C instead of 30° C (the standard condition, YES 3% glucose and 30° C) allows fission yeast to grow with longer generation times. At 35° C, fission yeast cells grow faster, with 20% reduction in generation time (2 h instead of the usual 2.5 h) [13]. Thus, eIF2α.S52A and WT cells were cultured in low-glucose conditions (0.1% glucose) at 30° C, as well as in regular 3% glucose growth medium at 20° C or 35° C. We observed reduced ^35^S incorporation consistent with a reduced translation rate in both cell types growing in a low glucose concentration medium or at 20° C with 3% glucose concentration. However, ^35^S incorporation was higher at 35° C than at 30° C, consistent with an increased rate of protein synthesis (Figure 3A).

Next, we analyzed the effect of these conditions on *S. pombe* longevity. We observed extended lifespan in eIF2α.S52A cells during either low-glucose or low-temperature growth, similar to that of WT cells growing under standard conditions. Furthermore, both cell types showed premature chronological aging when cultivated at a higher temperature (Figure 3B). Altogether, our results demonstrate that there is an inverse relationship between translational activity and longevity: higher protein synthesis during active growth phases leads to a reduced lifespan, whereas conditions that decrease translation rate (low temperature and caloric restriction) lead to an extended lifespan.

Then, we wanted to see how cell longevity was affected by changing the glucose concentration present in the culture medium at different points during the exponential growth phase. When eIF2α.S52A cells were shifted from a 3% to 0.1% glucose YES medium during the exponential phase (up to 6 h of growth), they presented an extended lifespan (Figure 3C). However, when the glucose concentration shift took place when cells were entering stationary phase (10 h of growth or later), their longevity was similar to those cultured in normal (3% glucose) YES medium during the whole experiment. Conversely, when eIF2α.S52A cells growing in 0.1% glucose YES medium were shifted to a 3% glucose concentration between 0 and 8 h of exponential growth, they showed a reduced lifespan (Figure 3C). However, when eIF2α.S52A cells grew from 12-24 h in 0.1% glucose medium (entering the stationary phase) prior to a shift to 3% glucose medium, their longevity was similar to those growing in 0.1% glucose throughout the entire experiment. These results suggest that changes produced by physiological processes which take place during the exponential growth phase are crucial for cells chronological aging, and may be responsible for the observed differences in longevity between WT and eIF2α.S52A cells.

### Specific inhibitors of protein synthesis rescue premature aging of eIF2α.S52A mutant cells by decreasing translation

**Figure 4:**
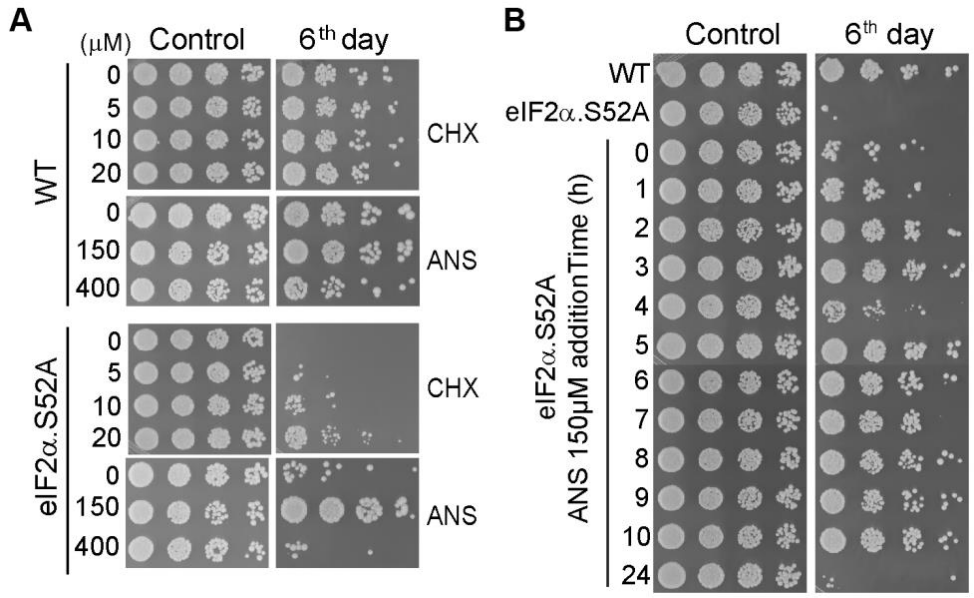
**Translation reduction by inhibitors of protein synthesis increases lifespan.** (**A**) Serial dilutions of WT and eIF2α.S52A cells growing in regular YES medium containing different sub-lethal concentrations of cycloheximide (CHX) or anisomycin (ANS) at the exponential phase (Control) or six days after reaching stationary phase, were plated onto YES agar plates and incubated at 30° C for 2-3 days. (**B**) Effect of ANS during the exponential growth phase. ANS (final concentration of 150 μM) was added to eIF2α.S52A cell cultures at the indicated time points during exponential growth. Serial dilutions of cells were plated as described in (**A**) and observed after six days of growth. Data information: (**A**, **B**) The results shown are representative of at least three independent experiments.

The above results indicated a negative correlation between translation rate and longevity under different physiological conditions. Thus, we reasoned that chemicals which specifically reduce translational activity should also have a positive effect on lifespan. To further demonstrate causality, we tested the longevity of WT and eIF2α.S52A cells after treatment with two different specific inhibitors of translation: cycloheximide (CHX) and anisomycin (ANS). First, we confirmed that sublethal concentrations of CHX and ANS have the capacity to reduce protein synthesis in both cell types (Supplementary Figure 1). Then we observed an extended lifespan in eIF2α.S52A cells cultivated in presence of 20 μM CHX or 150 μM ANS, showing a similar longevity to that of untreated WT cells (Figure 4A).

Interestingly, the positive effect of these translation inhibitors on fission yeast longevity was achieved only when the drugs were added during the early hours (up to 10 h) of the exponential growth phase (Figure 4B), consistent with results from Figure 3C showing growth under caloric restriction. Consequently, there is a window of opportunity to affect chronological aging in fission yeast, prior to entry into the stationary phase. Once cells pass this point, extension of lifespan was not possible in our experimental conditions.

### Neither glucose consumption nor intracellular ROS levels are altered in eIF2α.S52A mutant cells longevity assays

Several studies have demonstrated a strong relationship between glucose consumption, intracellular reactive oxygen species (ROS) levels and chronological aging [14, 15]. It is known that glucose cannot be detected in the extracellular medium for long after reaching stationary phase, suggesting that the type of metabolic processes taking place during exponential growth may condition chronological aging [15]. We first measured glucose consumption by WT and eIF2α.S52A cells in standard YES medium (3% glucose) during the exponential growth phase, showing that glucose was almost exhausted after 10 h of growth in both cultures (Supplementary Figure 2A). We also observed an increase in intracellular ROS levels when the cells entered stationary phase, as described before [16]. However, the increase in ROS levels was not significantly different between the WT and eIF2α.S52A cells (Supplementary Figure 2B). These results indicate that neither glucose consumption nor ROS levels differences are the cause of the reduced longevity of eIF2α.S52A cells seen in YES medium.

### RNA-seq analysis demonstrates differential translational-dependent gene expression in wild type and eIF2α.S52A cells

We have demonstrated the relationship between a slow-down of global translation rates and cellular longevity. However, those experiments did not exclude additional effects on aging promoted by eIF2α phosphorylation through the targeted translational activation of selected genes. Therefore, using next-generation sequencing (NGS), we studied whether eIF2α phosphorylation-dependent translational control promotes the up-regulation of specific genes which might also be involved in the aging process. We obtained total and ribosome-bound mRNAs from WT and eIF2α.S52A cells after 6 h of exponential growth, the point in time coinciding with the moment we observed significant differences in translation rates between both cell types. Furthermore, this was the “window” in which we have demonstrated that the aging fate of the culture is determined (Figures 3C, 4B).

RNA-Seq (RNA sequencing) results showed a good Pearson correlation among analyzed samples, with very little dispersion between WT and eIF2α.S52A cells at 0 h and 6 h (Supplementary Figure 3A). The fact that there were no significant differences in the mRNA composition of samples obtained from both strains at the beginning of the experiment indicates that eIF2 phosphorylation had little or no effect on specific mRNA translation at this time point, when cells are unstressed and growing exponentially. However, although we also observed a good correlation after 6 h of growth, there was a slightly higher dispersion of the data, suggesting that eIF2α phosphorylation already present at this time (Figure 1A) may have an effect on translation of specific mRNAs after several hours of exponential growth. Furthermore, we compared total mRNA in both WT and eIF2α.S52A cells at the same time points, showing a very good correlation between both strains at 0 h and a major dispersion after 6 h of growth (Supplementary Figure 3B). These changes may be due either to the fact that specifically translated mRNAs are an important part of the total mRNA, or because there may be side effects of transcriptional regulation. Then, the data presented above (Supplementary Figure 3A) were (data) displayed in graphs comparing 0 h and 6 h for each strain (Supplementary Figure 3C). The dispersion of the data was higher when time points where compared than when strains (at the same time point) were compared, indicating more changes in gene expression due to growth conditions (0 h to 6 h) than to genetic background of the strains assayed (WT vs. eIF2α.S52A).

**Figure 5:**
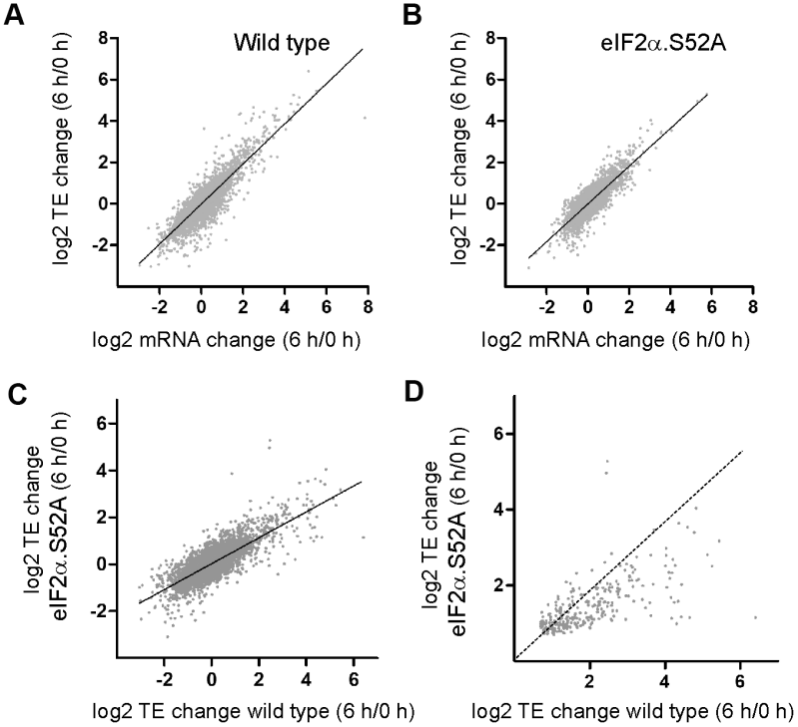
**Translation-dependent changes of gene expression mediated by eIF2α phosphorylation during the pre-stationary phase of growth.** (**A**, **B**) Scatter plots comparing log_2_ fold change in total mRNA levels and in translation efficiencies (TE) of (**A**) WT and (**B**) eIF2α.S52A cells growing exponentially (0 h) and after 6 h of growth in YES medium. (**C**) Scatter plot comparing log_2_ fold change in TE between WT and eIF2α.S52A cells growing exponentially (0 h) and after 6 h of growth in YES medium. (**D**) Same as in (**C**), but restricted to the group of genes whose translation efficiency increased in WT as well as in eIF2α.S52A cells. Dotted line indicates equal values in both strains. Data information: (**A**–**C**) The best fitting line of the data is shown. R^2^ (**A**) = 0.7605; R^2^ (**B**) = 0.6947; R^2^ (**C**) = 0.5977.

Next, we analyzed translational control of gene expression in the presence and absence of eIF2α phosphorylation. The number of sequence reads normalized to mRNA levels in the polysome fraction provides a good estimation of efficiency of translation (TE). Scatter plots comparing TE and total mRNA changes in both cell types are shown (Figure 5A, 5B). In both cases, the change in total mRNA when cells were entering the stationary phase was very similar to the observed translational change. However, there were specific genes which were more actively translated.

We were interested in genes that were translationally up-regulated by at least 50% after 6 h of growth (a TE change higher than 1.5-fold and p<0.05). We identified more up-regulated genes in WT cells compared to eIF2α.S52A cells (643 and 347 genes, respectively) (Figure 5C and Supplementary Tables 1, 2, 7, 8), likely because of activation of eIF2α phosphorylation-dependent pathways. Additionally, in the group of mRNAs that increased their translation efficiency in both types of cells, some of them (at least 96) presented a much higher translational activation in WT cells (Figure 5D and Supplementary Table 9).

Previous studies have shown that most genes regulated by eIF2α phosphorylation carry one or more uORFs located in the 5′-leader sequence of the mRNA, such as *ATF4* in mammals or *GCN4* in budding yeast [17, 18]. Thus, we searched for uORFs (ATG-dependent) in the up-regulated genes in both types of cells, finding that the proportion of genes containing ATG-dependent uORFs in WT cells was within the expected range with respect to that described in *S. pombe* (Pombase, https://www.pombase.com). However, cells lacking capacity for eIF2α phosphorylation had a lower proportion than expected (p<0.0132), which strongly suggests that under stress conditions, eIF2α phosphorylation regulates mRNA translation in the fission yeast *S. pombe* through uORFs (Supplementary Table 3).

**Figure 6:**
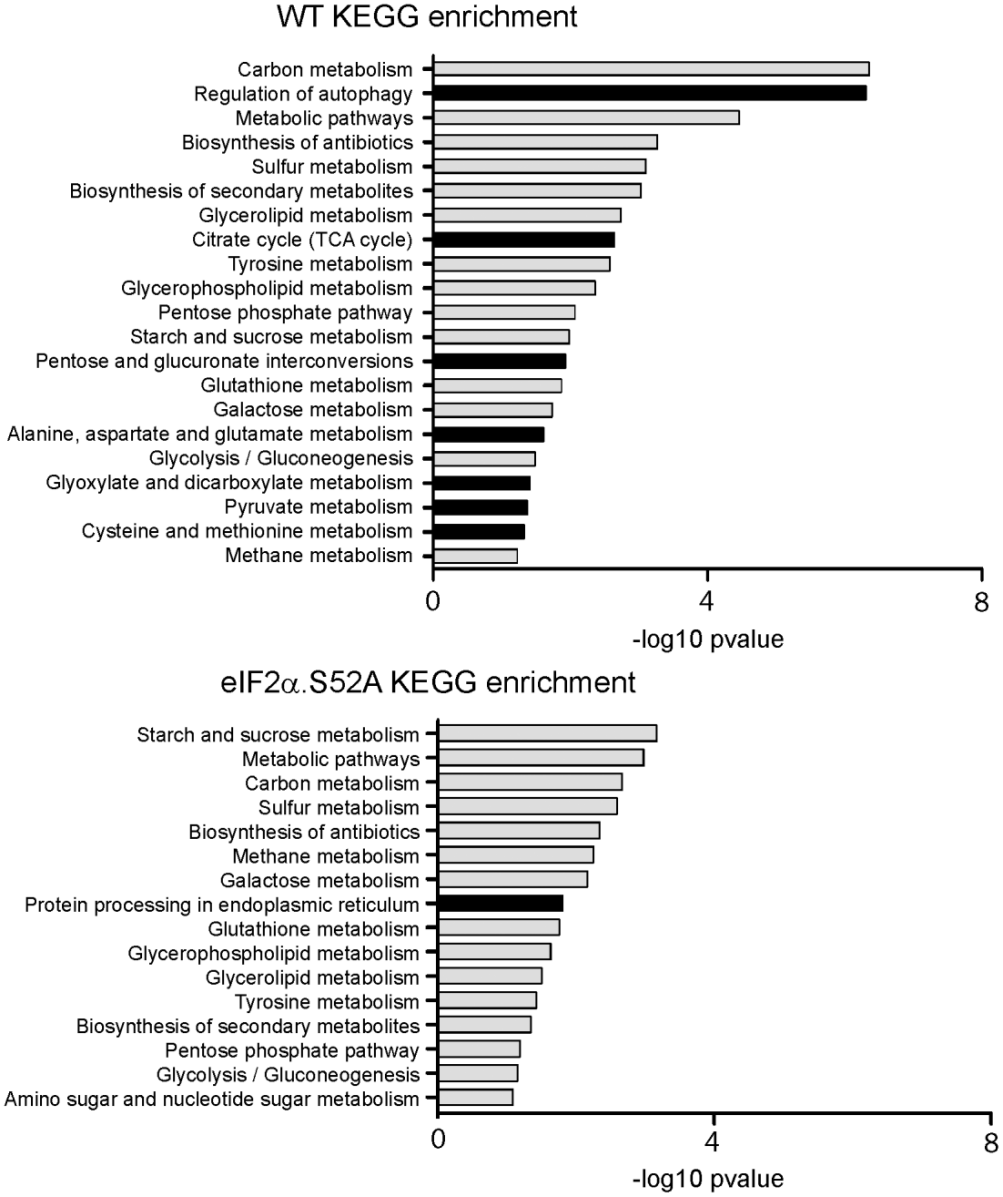
**KEGG analysis of RNA-Seq data.** Kyoto Encyclopedia of Genes and Genomes (KEGG) pathway enrichment analysis for up-regulated genes in wild type (WT) and eIF2α.S52A cells, according to RNA-Seq data. Dark bars denote categories enriched only in one type of cells.

RNA-Seq data were further analyzed by statistical analysis and performing enrichment pathways by using Gene Ontology (GO) and Kyoto encyclopedia of genes and genomes (KEGG) analysis. Gene ontology (GO) was applied to identify biological attributes for up-regulated and down-regulated genes found in the RNA-Seq data. They were classified into different functional categories according to the GO term enrichment analysis for Biological Process (Supplementary Tables 4–6). Comparing both types of cells, we observed that the most significantly up-regulated GO categories in WT cells were related to catabolic processes (*GO:0009056*), autophagy (*GO:0006914*) or cellular responses to toxic substances (*GO:0097237*). Furthermore, many of them were not only up-regulated in WT cells, but also were down-regulated in eIF2α.S52A cells. Conversely, these results showed an underrepresentation of genes related to ribosomal subunit biogenesis (*GO:0042273; GO:0042274*) and translation (*GO:0006412*), which were more prominent in the absence of eIF2α phosphorylation. In agreement with these results, KEGG pathway analysis confirmed that the up-regulated genes of WT cells were also enriched in the regulation of autophagy pathways, which was absent in cells unable to phosphorylate eIF2α (Figure 6 and Supplementary Tables 10, 11). Other pathways exclusively up-regulated in WT cells are related with very relevant metabolic processes, from the energy production point of view (citrate cycle and pyruvate metabolism), and also those related with the metabolism of certain amino acids (cysteine, methionine, alanine, aspartate, glutamate).

Altogether, these results underline the relevance of specific translational control of gene expression by eIF2α phosphorylation in the adaptive response of cells to stress, and especially the increased translation of autophagy-related proteins during the period leading up to the stationary phase of growth, which could play a critical role on the aging process.

### The onset of autophagy is delayed in eIF2α.S52A cells

**Figure 7:**
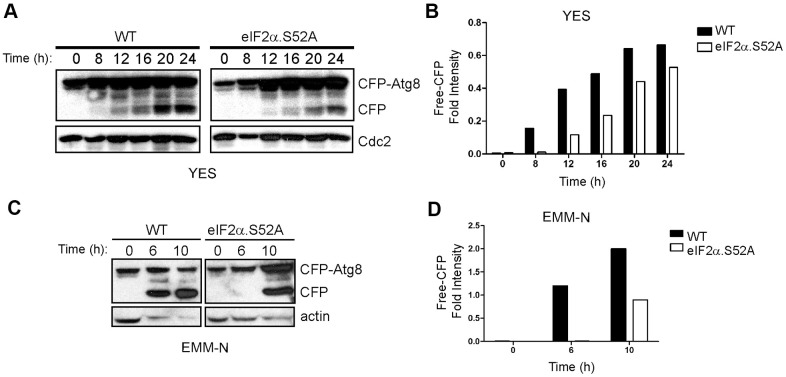
**Delay of autophagy onset in eIF2α.S52A cells during the aging process.** (**A**) WT and eIF2α.S52 values were normalized A cells expressing CFP-Atg8 were grown in YES medium and harvested at different time points. Cell extracts were analyzed by immunoblotting using antibodies against CFP and Cdc2 (loading control). (**B**) Quantitative analysis of CFP-Atg8 cleavage in panel (**A**). The intensity of CFP band was quantified and the values normalized using Cdc2 as loading control. (**C**, **D**) Similar experiments were performed with cells grown in minimal medium without nitrogen (EMM-N). Actin was used as loading control. Data information: (**A**–**D**) Shown results are representative of at least three independent experiments.

Our RNA-Seq results support the previously described relationship between autophagy and longevity, which establishes autophagy as a key determinant of cellular homeostasis maintenance, influencing lifespan and longevity (reviewed in [19, 20]). As seen in Supplementary Table 6 and Figure 6, the expression of autophagy-related genes was enriched in WT but not in eIF2α.S52A mutant cells. Thus, we considered the autophagy process as a good candidate to link eIF2α phosphorylation, translation and longevity. Autophagy activity was analyzed in cell cultures of both cell types entering stationary phase, using the cyan fluorescent protein (CFP)-Atg8 processing assay that is common in autophagy studies on fission yeast [21]. This assay is based on the fact that CFP-Atg8, which attaches to the inner membrane of the autophagosome, is delivered to the vacuole and processed upon induction of autophagy. As a consequence, a protease-resistant free CFP is generated that can be detected by western blot [22]. WT and eIF2α.S52A cells expressing an N-terminal CFP-tagged version of Atg8, were incubated for up to 24 h in rich media at 30° C (Figure 7A, 7B). In WT cells, we detected a free-CFP band after 8 h of growth, whereas appearance of this proteolytic product was delayed by about 4 h in eIF2α.S52A cells.

It is established that nitrogen depletion is a main inducer of autophagy in fission yeast [23]. Thus, in order to more accurately study the autophagy activity in both WT and eIF2α.S52A cells, a similar assay was performed in minimal medium without nitrogen (EMM-N). In this medium, nitrogen starvation occurs immediately in physiological synchrony, and autophagy should take place earlier than in YES medium. A CFP band had already appeared after 6 h of nitrogen starvation in WT cells and, again, a 4-hour delay was observed in eIF2α.S52A cells (Figure 7C, 7D), indicating an eIF2α phosphorylation-dependent onset of autophagy activity. These experiments demonstrate that autophagy is affected by the alteration in the translational initiation machinery and may be responsible for some of the effects in lifespan caused by eIF2α phosphorylation.

## DISCUSSION

Cell survival is enhanced by careful maintenance of proteostasis, including many mechanisms that are commonly established to reduce translation, such as TOR and the insulin-insulin like growth factor 1 inhibition pathways. Besides, another pathway which also reduces global translation rate in response to a variety of stresses and is considered the core of the integrated stress response (ISR), is phosphorylation of initiation factor 2 [8]. Considering entry into the stationary phase is not only fundamental to chronological aging but also is a stressful situation for the cell, here, we studied how phosphorylation of eIF2α affects cell longevity. eIF2α phosphorylation increased over time during the exponential growth phase, reaching a high level when cells entered the stationary phase. This is likely a consequence of the progressive deficit in essential nutrients which occurs as cells grow and deplete resources very quickly during exponential growth, given that eIF2α kinases are known to be activated under conditions of low glucose or nitrogen sources, such as NH_3_ or amino acids [9, 10]. Our analysis of chronological aging revealed a remarkable decrease of longevity in cells unable to significantly reduce global translation by phosphorylation of eIF2α. Given the dual function of eIF2α phosphorylation in the regulation of protein synthesis, its role in aging may be accomplished in two different but complementary ways: i) by reduction of global protein synthesis; and ii) by induction of gene-specific translation. Our results show that cells incapable of phosphorylating eIF2α presented higher levels of general protein synthesis than WT cells during exponential phase growth. However, protein synthesis decreased in both types of cells after 10 h of growth, coincident with the reduction of the growth rate as a sign of the entry into stationary phase. These results suggest that a decrease of translation by eIF2α phosphorylation while cells are actively dividing could have a positive effect on cell lifespan. However, once the cells decreased their metabolism in preparation for stationary phase, global translation rate was reduced in an eIF2α phosphorylation-independent manner.

The evident correlation between the reduction of global translation mediated by eIF2α phosphorylation and extended lifespan is supported by aging assays performed under conditions that slowed cell metabolism, including growth in low-glucose medium or at a sub-optimal growth temperature. In both situations, the sharp reduction of protein synthesis resulted in an increase of cell longevity, suggesting that a decrease in global translation during the exponential phase is important to allow fast-growing cells to reign in their nutrient usage and prepare for survival during the stationary phase.

An alternative explanation could be that the slow general metabolism, including protein synthesis, of cells growing in these conditions makes possible a better adaptation (i.e. protein homeostasis) in which eIF2α phosphorylation-dependent reprogramming of gene expression results less critical.

The mechanisms leading to translation reduction in these conditions remain (mechanisms) to be elucidated. One signaling pathway proposed to increase chronological lifespan in fission yeast by caloric restriction has been the TOR pathway through Sck2 (homolog to Sch9 in *S. cerevisiae* or S6K in mammals) regulation [14, 24]. Moreover, we cannot discard alternative translational regulation of CLS by activation of specific pathways. For instance, the cAMP/PKA pathway is activated when cells detect lack of glucose in the medium, promoting expression of specific genes involved in the stress response as well as in the adaptive response to oxidative stress that enhances CLS in a Sty1-dependent manner [15]. Altogether, these mechanisms may be responsible for the lifespan extension observed under low glucose conditions in cells lacking the ability to phosphorylate eIF2α. Moreover, a trend has been described for lower temperature being associated with longer lifespan [25]. Previous studies in different organisms support this approach (reviewed in [26]); however, the mechanisms which regulate this response still remain unknown. The complementary trend, that organisms age faster at higher temperatures [27] is supported by our results in faster-growing cells at 35° C [13], presenting accelerated translation rates and consequently, a reduced lifespan. In this case, the general fast metabolism, including protein synthesis, may negatively affect the establishment of adaptation mechanisms, promoting irreversible changes in cell homeostasis, in general, and specifically in proteostasis, that finally lead to premature aging.

To further confirm the causality between translation rate and aging, we performed longevity assays in the presence of sublethal concentrations of specific inhibitors of protein synthesis (CHX and ANS), reversing the longevity deficiency of eIF2α.S52A cells resulting in a lifespan similar to that of WT cells in normal conditions. Moreover, these only had a positive effect on lifespan when applied during the exponential growth phase. Our results confirm a mechanism where reduction of translation during exponential growth has a positive effect in longevity several days after. Perhaps, some irreversible changes happen when translation is not tuned down, producing aging signal or non-reparable damage in the cell.

Protein synthesis decreased in both types of cells when they entered stationary phase, independently of eIF2α phosphorylation. This period coincided with an increase in intracellular ROS levels, which reached maximum levels at the beginning of the stationary phase, as described before [16]. Again, no significant differences were observed between WT and eIF2α.S52A cells. A recent study has described that the oxidative stress response associated with protein synthesis is mainly regulated by phosphorylation of eukaryotic elongation factor 2 (eEF2) [28]. In this case, we could hypothesize that an accumulation of ROS species promotes a down-regulation of translation mediated by eEF2. A progressive decrease of glucose concentration in the extracellular medium was observed during the exponential phase, reaching almost undetectable levels upon entry into stationary phase. No differences in glucose consumption were detected between WT and mutant cells, and it is known that low glucose concentrations can lead to activation of nutrient-sensing translation regulation mechanisms [15, 29]. Our results indicate that these mechanisms are independent of eIF2α phosphorylation and therefore are not responsible for the early aging phenotype of eIF2α.S52A cells.

All these results establish a window of opportunity to affect chronological aging in fission yeast prior to entry into stationary phase, and demonstrate that the effect on proteostasis mediated by eIF2α phosphorylation meets the requirements of the “hallmarks of aging” described previously [30].

Simultaneously to a decline in global protein synthesis, selected mRNAs encoding proteins required for cellular maintenance, repair and turnover pathways showed an increase in translation upon eIF2α phosphorylation. Many studies have shown a correlation between enhanced lifespan and overproduction of specific mRNAs. Moreover, it has been demonstrated that the changes of age-related proteins detected at the proteome level are caused by post-transcriptional mechanisms, including selective translation, since the expression of 77% of the age-associated proteins were not linked to expression of the corresponding transcripts [31].

The best-studied examples of genes selectively translated in an eIF2α phosphorylation-dependent manner are *ATF4* in mammals and *GCN4* in budding yeast. Translation of both genes is up-regulated by eIF2α phosphorylation, which then promote transcription of genes involved in the stress response. The fission yeast *S. pombe* lacks *ATF4* and *GCN4* orthologs, although similar regulation of expression of *fil1* has been recently described, which is responsible for transcription of other genes involved in the general amino acid response [32]. An RNA-Seq analysis was performed with the aim of studying the effect of the eIF2α phosphorylation during the early stages of cell growth and its implications in the aging process. Our results showed that those genes upregulated in eIF2α phosphorylation-competent cells were enriched mainly in Gene Ontology categories related to autophagy, catabolic processes and response to toxic substances and stimuli. These results are consistent with the fact that phosphorylation of eIF2 is one of the main responses to various stresses. Furthermore, in catabolic processes, the cell obtains energy through degradation systems such as autophagy. The RNA-Seq results also showed a down-regulation of categories related to ribosome subunit biogenesis or translation, consistent with the reduction of global translation rate in the presence of eIF2α phosphorylation. Among the up-regulated genes related to autophagy activity, *atg1*, *atg2* and *atg7* have been described to be required for extended lifespan [33]. These results support our hypothesis of a relationship between autophagy and eIF2α phosphorylation, as well as its positive role in cell lifespan. This idea reconciles, at least in part, a previous report by Tyler’s group where GCN4-dependent induction of autophagy during global shutdown of protein synthesis could be an unifying molecular mechanism for many interventions that extend replicative lifespan in budding yeast [34]. Moreover, our RNA-Seq analysis shows very similar results of translational gene expression activation between eIF2α phosphorylation and *fil1* (a downstream effector of eIF2α kinases in *S. pombe*) translation [32]. Other up-regulated genes are involved in the cAMP/PKA nutrient signaling pathway, such as *pcr1* or *git5*, which, together with autophagy, is one of the main pathways that plays an important role in aging.

Maintenance of cellular proteostasis depends not only on protein synthesis but also on degradation systems. In this context, autophagy clears damaged organelles, misfolded proteins and aggregates which accumulate as cells age. Many nutrient-sensing longevity-associated pathways, like TOR and IGF-1 inhibition, require autophagy components in order to confer longevity [35]. Moreover, the ISR can regulate cell survival through activation of autophagy [8]. However, precise mechanisms by which eIF2α phosphorylation contributes to autophagy induction are still poorly understood, although it has been reported that the eIF2α/ATF4 pathway is essential for stress-induced autophagy gene expression [36]. We demonstrated that cells present a delay in onset of the autophagy process in the absence of eIF2α phosphorylation. Indeed, previous studies have demonstrated that the absence of eIF2α phosphorylation and the delayed onset of autophagy correlate with the delayed entry into the G1 phase induced by ammonium deprivation in *S. pombe* [10], which in turn may be relevant for cell longevity.

In conclusion, the results obtained in this work provide a new framework for regulation of cellular aging mediated by phosphorylation of eIF2α. On one hand, a reduced global protein synthesis rate caused by this phosphorylation leads the cell to improve efficiency of energy utilization as well as to avoid the overloading of protein folding and degradation systems in order to maintain cellular proteostasis. On the other hand, eIF2α phosphorylation favors translation of selected genes involved in stress response pathways or in activation of other aging-related processes, such as autophagy. The role played by some of these genes in cellular lifespan should be subjected to future investigation. Both effects of eIF2α are not mutually exclusive, occurring dynamically in the cells for the promotion of cellular longevity. In fact, our results suggest that events that take place during the exponential phase of growth promote the phosphorylation of eIF2α, leading to both a reduction in global translation and an increase in specific gene expression, which result critical for lifespan extension.

## MATERIALS AND METHODS

### Yeast strains and growth conditions

The *S. pombe* strains used in this study are listed in Table 1. Yeast growth media and general methods were as described previously [37]. Cells were grown at 30° C in rich medium with supplements (YES, Formedium) or in Edinburgh minimal medium (EMM). For growth under glucose starvation conditions, cells were pre-cultured in rich medium containing 3% glucose (YES), and then transferred to the same medium containing 0.1% glucose.

**Table 1:** *S. pombe* strains.

**Name**	**Genotype**	**Source**
WT	*972 h^-^*	Paul Nurse
eIF2α.S52A	*eIF2alfa.S52A:Ura4 ura4-D18 h^-^*	E. Boye
Hri1Δ	*hri1::ura4^+^ ura4-D18 h^+^*	S. Moreno
Hri2Δ	*hri2:: ura4^+^ ura4-D18 h^+^*	S. Moreno
Gnc2Δ	*gcn2::ura4^+^ ura4-D18 h^-^*	S. Moreno
Hri1Δ Hri2Δ	*hri1::ura4^+^ hri2::ura4^+^ ura4-D18 h^-^*	J.J. Berlanga
Hri1Δ Gcn2Δ	*hri1::ura4^+^ gcn2::ura4^+^ ura4-D18 h^-^*	J.J. Berlanga
Hri2Δ Gcn2Δ	*hri2::ura4^+^ gcn2::ura4^+^ ura4-D18 h^+^*	J.J. Berlanga
Hri1Δ Hri2Δ Gcn2Δ	*hri1::ura4^+^ hri2::ura4^+^ gcn2::ura4^+^ ura4-D18 h^+^*	J.J. Berlanga
Wis1DD	*wis1DD:12myc:Ura4 leu1-32 ura4-D18 h^-^*	P. Russell
Wis1DD S52A	*eIF2alfa.S52A:Ura4 wis1DD:12myc:Ura4 ura4-D18 leu1-32 h^-^*	This study
Rsv2	*rsv2::kan^R^ leu1+ ura4^+^ ade6^+^* *h^-^*	This study
WT atg8:CFP	*CFP-atg8::leu1 leu1^+^ ura4^+^ h^-^*	Li Lin Du
Rsv2 atg8:CFP	*CFP-atg8::leu1 rsv2::Kan^R^ leu1^+^ ura4^+^*	This study
eIF2α.S52A:CFP	*CFP-atg8::leu1 eIF2α.S52A:Ura4 leu1^+^ ura4^+^ h^-^*	This study
WT pLB-DBlet	*972 leu1-32 ura4-D18 + pLB-Dblet*	This study
WT prsv2	*972 leu1-32 ura4-D18 + pLB-Dblet rsv2*	This study
S52A pLB-DBlet	*eIF2α.S52A:Ura4 leu1-32 ura4-D18 + pLB-Dblet*	This study
S52A prsv2	*eIF2α.S52A:Ura4 leu1-32 ura4-D18 + pLB-Dblet rsv2*	This study
Rsv2Δ pLB-DBlet	*rsv2::kan^R^ leu1^-^ ura4^+^ ade6^+^ + pLB-Dblet*	This study
Rsv2Δ prsv2	*rsv2::kan^R^ leu1^-^ ura4^+^ ade6^+^ + pLB-Dblet rsv2*	This study

### Stationary phase conditions and survival assays

Fission yeast cells were grown at 30° C in YES medium until they reached late stationary phase (OD_550_ = 5-9, approximately) depending on the strains and growth conditions. Identical numbers of cells from cultures at exponential phase (OD_550_ = 0.5-1) or several days after reaching stationary phase, were inoculated into the first column of a 96-well plate. Serial dilutions (1/5) were prepared across the 96-well plate, then spotted on duplicate YES agar plates using a 48-pin tool (VP 407 AH from VP Scientific). Plates were incubated for 2-3 days at 30° C.

### Electrophoresis and immunoblotting

Protein extracts (100 μg) were separated by sodium dodecyl sulfate polyacrylamide (40% acrylamide/bis 19:1, BioRad) gel electrophoresis (SDS-PAGE) and transferred onto polyvinylidene difluoride (PVDF) membranes (Immobilon-P; Millipore). For immunoblots, the antibodies used at a 1/1000 dilution were rabbit anti-eIF2α (Santa Cruz), rabbit anti-eIF2α-P (Cell Signaling), mouse anti-cdc2 (Abcam), mouse anti-actin (MP Biomedicals) or mouse anti-GFP (Roche), followed by anti-rabbit or anti-mouse secondary antibodies conjugated to horseradish peroxidase (Promega), used at a 1/5000 dilution. After extensive washing, the immunoreactive bands were detected using the Immobilon Crescendo Western HRP substrate (Millipore).

### Separation of polysomal RNA by sedimentation centrifugation and total RNA extraction

Cells in exponential growth phase (OD_550_ = 0.5-1) were processed and loaded onto sucrose gradients as described in [38], without adding heparin. Gradients were centrifuged in a Beckman SW40 rotor at 39000 rpm for 135 min at 4° C and fractionated in an ISCO Density Gradient Fractionator while monitoring absorbance at 254 nm. RNA was isolated from polysomal fractions by treatment with 1 volume of phenol:chloroform:isoamyl alcohol (25:24:1). After centrifugation, the aqueous phase was washed with chloroform and RNA was precipitated with 1 volume of isopropanol and 0.1 volumes of 3 M sodium acetate at -20° C overnight, washed with 70% ethanol and resuspended in RNase free water. For total RNA extraction, 10 ml of exponential growth phase (OD_550_ = 0.5-1) yeast culture was harvested and processed as described in [38].

### Metabolic labeling of proteins

Yeast cells in the exponential growth phase (OD_550_ = 0.5-1; about 10^6^-10^7^ cells) were pelleted (3000 x g for 5 min at room temperature) and resuspended in 500 μl of YES medium. Then, cells were pulsed for 15-20 min with 2 μl of 10 mCi/ml [^35^S]-Met/Cys (ProMix, Amersham) at 30° C. After centrifugation (14000 x g for 1 min) at 4° C, cells were resuspended in cell lysis buffer (PBS containing 1% Triton X-100; 0.1% SDS; 1 mM PMSF (Phenylmethylsulfonyl fluoride)/Complete^TM^ Mini EDTA-free Protease Inhibitor Cocktail, Roche) and disrupted with glass beads for 15 min in a vortex. Cell extracts were centrifuged at 4° C for 20 min at 14000 x g. Proteins were analyzed by 10% SDS-PAGE, fixed in 7.5% acetic acid/25% ethanol for 15 min and stained with Coomassie staining solution (50% methanol; 0.1 Coomassie Blue G-250; 7% acetic acid) for 30 min at room temperature. Finally, gels were subjected to fluorography in 1 M salicylate for 45 min, dried and exposed to x-ray film (Agfa Curix RP2 Plus films).

### Measurement of glucose concentration in the extracellular culture media

In order to measure the glucose concentration of cell culture supernatants, 1 ml samples of growing cultures were harvested at different times during exponential growth phase and after reaching stationary phase, pelleted and the supernatant stored at -70° C. Glucose measurement was carried out employing a commercial spectrophotometric assay kit (Cromatest®, Linear Chemicals).

### Measurement of intracellular H_2_O_2_ levels

Relative intracellular peroxide levels were analyzed using the redox-sensitive fluorescent probe 2′, 5′-dichlorofluorescein diacetate (DCFH-DA, Molecular Probes), which produces green fluorescence in the presence of H_2_O_2_ in both living and dead cells. In order to distinguish living from dead cells, propidium iodide (PI) was used as a secondary indicator dye. 1 ml of cells growing in rich medium was harvested at different times and incubated with 10 μg/ml DCFH-DA for 40 min at 30° C. Cells were centrifuged at 14000 x g and washed twice with citrate buffer (50 mM sodium citrate, pH 7.0). The pellets were then resuspended in an appropriate volume of citrate buffer containing 10 μg/ml PI. Peroxide steady-state levels and cell viability were then analyzed by flow cytometry. PI was monitored in channel FL3 (red fluorescence-detecting), whereas DCFH-DA was monitored in channel FL1 (green fluorescence-detecting) in a FACS Canto A (Becton Dickinson) cytometer. Only cells negative for PI staining were analyzed for DCFH-DA-dependent green fluorescence. A total of 10000 PI-negative cells were analyzed for each strain and experimental condition. The flow cytometry data were further analyzed using FlowJo V10 software.

### CFP-Atg8 processing autophagy assay

For nitrogen deprivation experiments, cell cultures were shifted from growth medium (EMM) to a nitrogen-free medium (EMM-N). For aging experiments, cells were grown in rich medium (YES). Cell lysates were prepared in lysis buffer (8 M urea; 100 mM NaH_2_PO_4_; 50 mM Tris-HCl pH 8.0) and resolved by 10% SDS-PAGE and immunoblotted with an anti-GFP antibody (1/1000 dilution, Roche).

### RNA-Sequence analysis

A total of 16 RNA samples were prepared for deep sequencing (RNA-Seq) by a specialized company (Macrogen). For library construction, total and polysomal RNA was extracted. After passing quality control, samples were processed for library construction. The sequencing library was prepared by random fragmentation of the cDNA sample, followed by 5′ and 3′ adapter ligations. The library kit used was TruSeq Stranded mRNA LT Sample Prep kit (Illumina) following the TruSeq Stranded mRNA Sample Preparation Guide, Part #15031047 Rev. E. RNA-Seq was carried out on an Illumina HiSeq 2500 platform. Paired-end reads were sequenced with a length of 100 nucleotides and FASTQ raw data were generated utilizing Illumina package bcl2fastq (v1.8.4). Raw reads, in FASTQ format, were quality checked with FASTQC [39]. For each sample, paired-end reads were aligned against *S. pombe* genome (ASM294v2 Ensembl fungi release 35) and sorted by coordinate position with STAR [40]. Alignment files (BAM) were indexed with SAMtools [41] and visualized with Integrative Genomics Viewer (IGV) [42, 43]. Aligned reads were assigned to *S. pombe* genes with function QC from QoRTs software package [44], using default parameters for paired-end and strand-specific sequences. Normalization of read counts and differential expression analysis were carried out with bioconductor package DESeq2 [45] using default parameters. A table containing both raw and normalized counts, log fold-changes, p values, adjusted p values and functional descriptions for each gene was prepared and sent to FIESTA Viewer [46] to perform additional filters.

For further analysis, only genes with p<0.05 were considered to perform a functional enrichment analysis based on KEGG [47] and “biological process” terms from Gene Ontology [48].

### Data availability

The RNA-Seq data from this publication have been deposited to the GEO and assigned the identifier GSE155256.

## Supplementary Material

Supplementary Figures

Supplementary Table 1

Supplementary Table 2

Supplementary Table 3

Supplementary Table 4

Supplementary Table 5

Supplementary Table 6

Supplementary Table 7

Supplementary Table 8

Supplementary Table 9

Supplementary Table 10

Supplementary Table 11
