# Supplementary material for: Translational control of gene expression by eIF2 modulates proteostasis and extends lifespan
Source: Aging (Albany NY). 2021 Apr 26;13(8):10989–1009. doi: 10.18632/aging.203018 (PMC8109070; doi:10.18632/aging.203018)
Supplement: Supplementary Figures [file aging-13-203018-s001.pdf]

SUPPLEMENTARY FIGURES

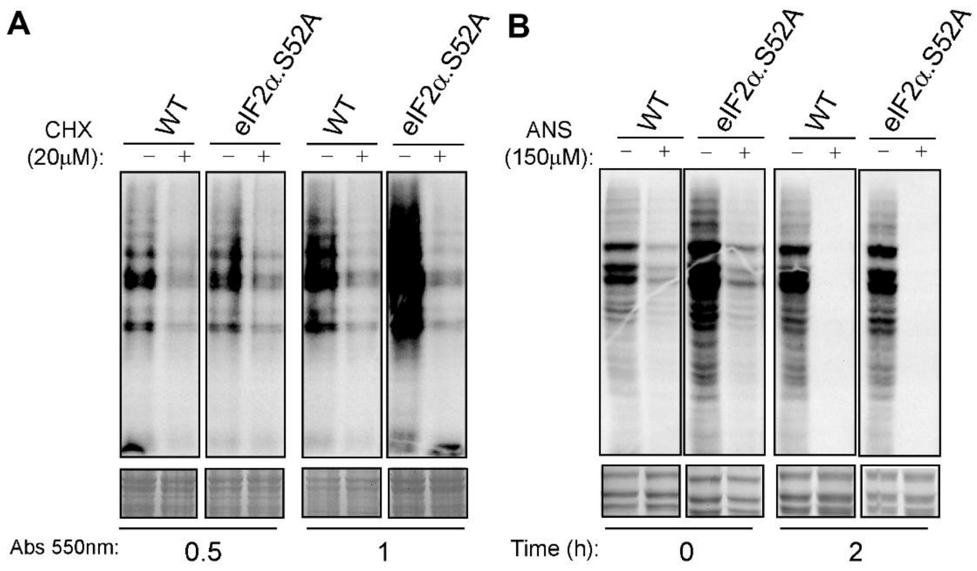

Supplementary Figure 1. Down-regulation of translation by specific inhibitors of protein synthesis. (**A**, **B**) Metabolic labelling of wild type (WT) and eIF2α.S52A cells grown in YES medium containing (**A**) 20 μM cycloheximide (CHX) or (**B**) 150 μM anisomycin (ANS) prior to labelling. Cells were cultivated to exponential growth phase (OD_{550} = 0.5, time 0 h) or later time points, labelled for 10 min and harvested. Labelled proteins present in cell extracts were visualized by autoradiography after SDS-PAGE separation (top panel). Coomassie staining shows the total protein levels loaded in each lane (bottom panel). Data information: (**A**, **B**) Shown are the results of an experiment of at least three independent experiments with similar results.

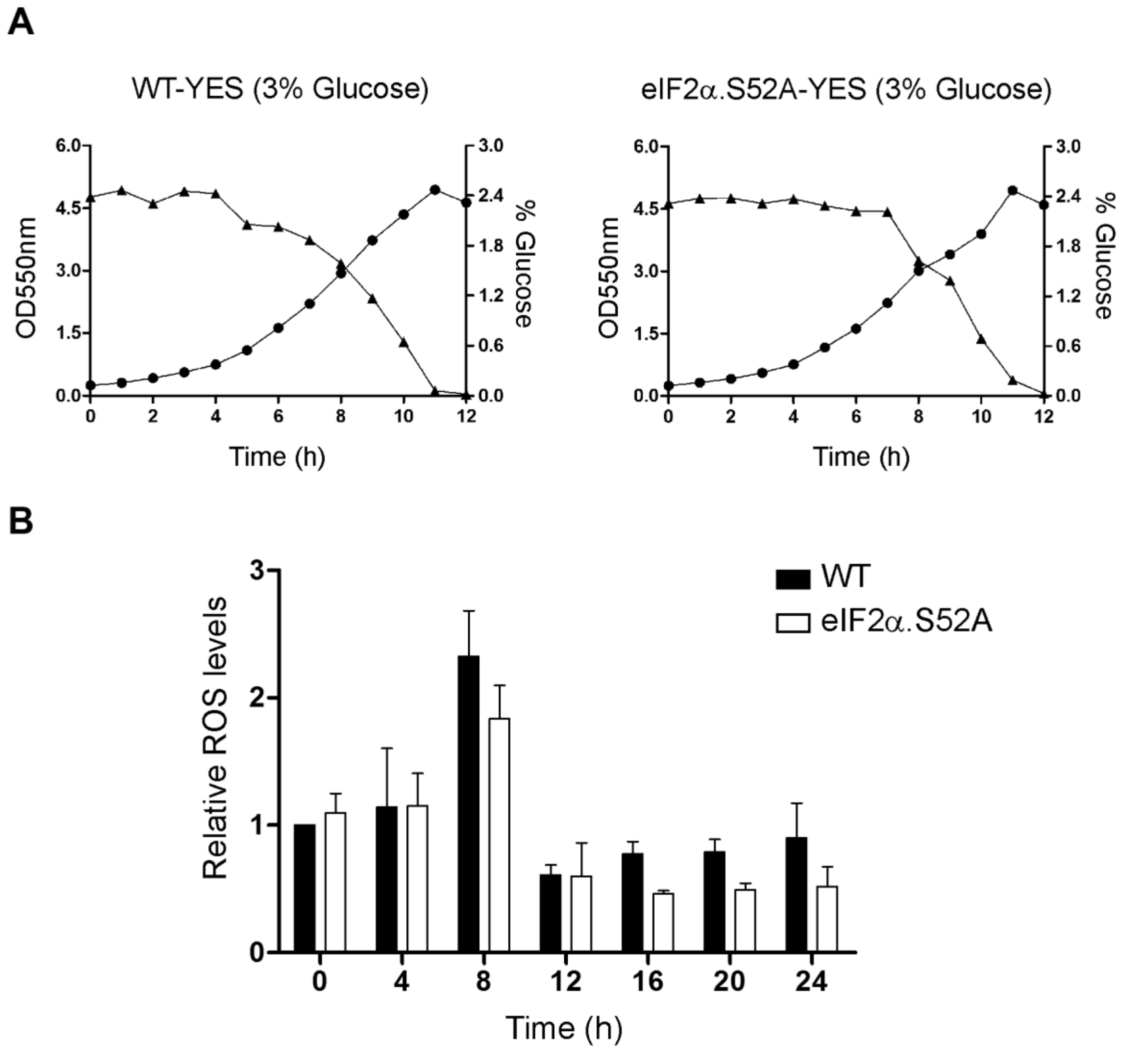

Supplementary Figure 2. Glucose consumption and intracellular ROS concentration are not altered in eIF2α.S52A cells during growth. (**A**) Growth curve (OD at 550 nm) and glucose consumption of wild type (WT) and eIF2α.S52A cells. Both strains were grown in YES medium and optical density (circles) and % of glucose in the extracellular medium (triangles) were measured during exponential growth phase. (**B**) Intracellular ROS levels of WT and eIF2α.S52A cells. Both strains cells growing in YES medium during exponential phase (0-10 h) and beyond (up to 24 h) were incubated with DCFH-DA and H_2O_2 levels were measured by flow cytometry. Error bars indicate the standard deviation (SD) of four independent experiments. No statistically significant differences were found at any time point.

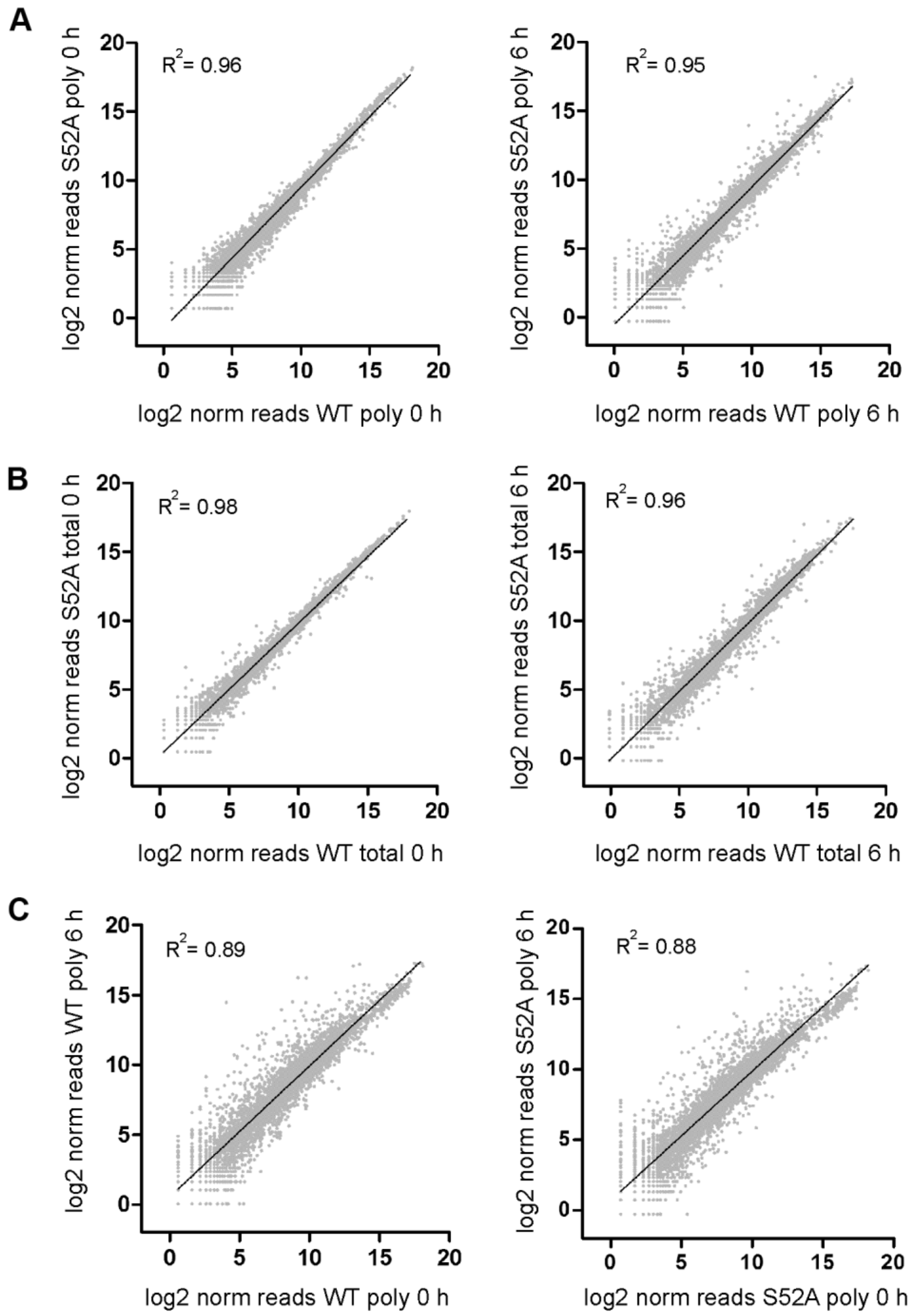

Supplementary Figure 3. Deep sequencing of wild type and eIF2α.S52A of total and polysomal mRNAs during the pre-stationary growth phase. (**A**) Scatter plots comparing mRNA levels from polysome (poly) fractions of wild type (WT) and eIF2α.S52A (S52A) cells growing exponentially (0 h) or after 6 h of growth in YES medium. (**B**) Same as in (**A**), but for the comparison between total mRNA levels in WT and eIF2α.S52A cells. (**C**) Same as in (**A**), but for the comparison between mRNA levels from polysome fractions of exponentially growing cells and after 6 h of growth in WT and eIF2α.S52A strains. Data information: (**A**–**C**) All data have been normalized to reads. The results of a single experiment are shown. Data information: (**A**–**C**) The best fitting line of the data is shown.
